# Editorial for the Special Issue “Current Research on Cancer Biology and Therapeutics”

**DOI:** 10.3390/ijms25052802

**Published:** 2024-02-28

**Authors:** Rafael Coveñas

**Affiliations:** Laboratory of Neuroanatomy of the Peptidergic Systems, Institute of Neurosciences of Castilla and León (INCYL), University of Salamanca, 37007 Salamanca, Spain; covenas@usal.es; Tel.: +34-923294400 (ext. 1856)

Cancer is a major health problem, in 2020 caused 10 million deaths and in 2040, 28,4 million patients suffering from the disease are expected [1]. One promising cancer research line is the search for compounds that specifically destroy tumor cells and show minimal side-effects. Antitumor compounds must specifically block cancer cellular events such as cell proliferation and survival, invasion, and metastasis as well as angiogenesis; mechanisms that are mediated by receptors, ligands, and signaling pathways which regulate the protein/genetic machinery of tumor cells. This Special Issue presents compounds exerting antitumor effects and increases the knowledge on potential and promising anticancer strategies to be used in the near future in clinical practice. These compounds include acetylcorynoline (for colon cancer) [2], BaP1 (a benzo[a]phenoxazine derivative, for colorectal cancer) [3], sarco/endoplasmic reticulum calcium ATPase inhibitors (for papillary thyroid carcinoma) [4,5], neuropeptide Y (for liver cancer, Ewing sarcoma, and cholangiocarcinoma) [6], neuropeptide Y receptor antagonists (for colorectal cancer and breast cancer) [6], and neurokinin-1 receptor antagonists (for different cancer types) [7]. These previous studies have opened new lines of research on targetable molecules to explore promising antitumor therapeutic strategies. The researchers participating in this Special Issue have also focused their studies on the non-apoptotic cell death mechanisms involved in the treatment of drug-resistant melanoma [8]; on the silencing of fatty acid elongase 4 and 6 genes, using small interfering RNAs, against colorectal tumor cells [9]; on the clinical importance of circulating microRNAs in the diagnosis, prognosis, and monitoring of pancreatic ductal adenocarcinoma [10]; and on the antitumor efficacy of oncolytic vaccinia viruses harboring lectins against hepatocellular carcinoma [11].

This Special Issue presents three antitumor strategies against colorectal cancer [2,3,9]. The group of Jang from the College of Korean Medicine (Seoul, Republic of Korea) has shown that acetylcorynoline, in a dose-dependent manner, blocked tumor cell growth by promoting apoptosis and cell cycle arrest in human colon cancer cells; decreased the protein expression of oncogenic genes; inhibited the expression of c-myc via MID1-interacting protein 1/CCR4-NOT transcription complex subunit 2; and, in combination therapy with low doses of 5-fluorouracil and doxorubicin (currently administered in clinical practice), acetylcorynoline exerted a synergic antitumor action [2]. This preclinical study demonstrated that the latter combination therapy decreased the side effects induced by chemotherapeutic agents and that the antitumor efficacy against colon cancer, which unfortunately shows a low survival rate, was greatly increased. Taken altogether, the data suggest that acetylcorynoline is a promising antitumor agent administered alone, but is especially effective as a combination therapy with chemotherapeutic agents. The second therapeutic strategy to treat colorectal cancer has been performed by Sousa and co-workers from the University of Minho (Braga, Portugal) [3]. They have tested the antitumor efficacy of a new benzo[a]phenoxazine derivative named BaP1 (N-(5-(4-ethoxy-4-oxobutyl) amino)-10-methyl-9H-benzo[a]phenoxazine-9-ylidene) ethanaminium chloride). This compound reduced the survival, proliferation, and migration of human colorectal cancer cells, and BaP1 blocked in vivo tumor growth and proliferation as well as angiogenesis. BaP1 promoted the formation of reactive oxygen species and was stored in lysosomes, leading to the permeabilization of the lysosome membrane, cytosol acidification, and the death of tumor cells by apoptosis. BaP1 is a promising antitumor lysosomal-targeting candidate, and the lysosome membrane permeabilization strategy is a promising therapeutic tool against one of the most mortal cancers, colorectal cancer, which additionally shows a high incidence. The third therapeutic strategy reported in this Special Issue against colorectal cancer, which showed increased fatty acid elongation, was the silencing of the fatty acid elongase 4 and 6 genes after using small interfering RNAs [9]. An increase in very-long chain fatty acids has been previously reported in the sera and tissues of patients suffering from colorectal cancer [12,13,14]. One of the studies reported in the Special Issue was performed by Czumaj and her group from the Medical University of Gdansk (Gdansk, Poland). An overexpression of the elongase 4 and 6 genes in human colorectal cells and tissues has been observed, and the authors demonstrated that small interfering RNAs for both mentioned genes blocked the elongation of fatty acids and decreased the proliferation/migration of human colorectal cells. Moreover, the authors reported that the silencing of the elongase 4 gene in normal colon cells did not affect its viability, but the viability of these cells was affected after the silencing of fatty acid elongase 6. The data suggest that the decrease in the survival of colorectal cancer cells was associated with alterations in the elongation mechanisms of fatty acids and hence fatty acid elongases are new promising antitumor targets against colorectal cancer.

Two papers in this Special Issue have focused on the use of sarco/endoplasmic reticulum calcium ATPase (SERCA) inhibitors for the treatment of sorafenib-resistant papillary thyroid carcinoma [4,5]. Both studies were performed by Park and co-workers from the Yonsei University College of Medicine (Seoul, Republic of Korea). Papillary thyroid carcinoma has a poor prognosis when resistance to antitumor drugs occurs. The expression of SERCA (favors calcium influx into the endoplasmic reticulum) is increased in sorafenib-resistant papillary thyroid carcinoma [4] and its activation blocks cytoplasmic calcium overload, leading to cellular resistance to apoptotic mechanisms and genotoxic stress promoted by the administration of sorafenib (a multi-targeted kinase inhibitor affecting tumor cell growth). The antitumor effect of SERCA inhibitors named 6-methoxy-2-[(4-methoxy-3,5-dimethylpyridin-2-yl) methylsulfinyl]-1H-benzimidazole and 2-tert-butylbenzene-1,4-diol against sorafenib-resistant papillary thyroid carcinoma cells was studied [4]. The authors found that, after the administration of these inhibitors, tumor shrinkage was increased in a xenograft model of human sorafenib-resistant papillary thyroid carcinoma and that the combination therapy of SERCA inhibitors and sorafenib promoted endoplasmic reticulum stress-mediated apoptotic mechanisms in human papillary thyroid carcinoma cells resistant to sorafenib. Importantly, the administration of SERCA inhibitors or sorafenib alone did not exert any effect in sorafenib-resistant papillary thyroid carcinoma. Moreover, the authors found similar results regarding other SERCA inhibitors (C_16_H_15_F_2_N_3_O_4_S; C_41_H_64_O_14_) in a second paper [5]. Both studies exemplify the targeting of antitumor drug-resistant papillary thyroid carcinoma by applying a new combination therapy and demonstrate the involvement of SERCA in the resistance to anticancer drugs such as sorafenib.

In this Special Issue, two reviews highlight the important role played by peptides in tumor development [6,7]. Both studies were performed by Coveñas and colleagues from the University of Salamanca (Salamanca, Spain). The first review was focused on the involvement of the neuropeptide Y peptide family in cancer and on the potential promising anticancer therapeutic strategies that could be applied, and the second review was devoted to the widespread antitumor action exerted by the drug aprepitant, a neurokinin-1 receptor antagonist exclusively used in clinical practice as an antiemetic. The first paper was focused on the involvement of neuropeptide Y, peptide YY, and pancreatic polypeptide in twenty-two different cancer types [6]. Tumor cell proliferation, migration, invasion/metastasis, and angiogenesis are mediated by neuropeptide Y receptors, the expression of which has been associated with lymph node metastasis, perineural invasion, advanced stages, and tumor growth and survival. Peptide YY inhibited cell growth and invasion/migration in some cancers (e.g., prostate, pancreas, liver, esophagus, colorectal, breast). Neuropeptide Y promoted angiogenesis and cancer cell growth, migration, and metastasis in some tumors such as pancreatic cancer, neuroblastoma, colorectal cancer, and breast cancer, whereas in others (e.g., liver cancer, Ewing sarcoma, cholangiocarcinoma) the opposite effect was favored; that is, it exerted an antitumor action. Previous data indicate that the neuropeptide Y peptide family is a peptidergic system with a high potential for cancer treatment; accordingly, the use of neuropeptide Y receptor antagonists or neuropeptide Y/peptide YY agonists targeting neuropeptide Y receptors are promising new antitumor strategies. The second review was focused on the role played by the substance P/neurokinin-1 receptor system in cancer and on the promising use of the neurokinin-1 receptor antagonist aprepitant as an antitumor agent, since this drug promoted apoptosis in many tumor cell types [7]. Many different cancer cells also overexpress the neurokinin-1 receptor, which is involved in the viability of these cells, and in addition, this overexpression could be used as a tumor biomarker for cancer diagnosis. Moreover, substance P, after binding to the neurokinin-1 receptor, favored tumor cell growth, migration, and invasion/metastasis as well as angiogenesis and also promoted an anti-apoptotic effect in many different tumor cells. The broad-spectrum antitumor action of the drug aprepitant against many different tumor types (e.g., neuroblastoma, retinoblastoma, breast cancer, pancreatic cancer, liver cancer, colorectal cancer, lung cancer) was reviewed and the authors suggested the urgent repurposing of this drug as an antitumor agent alone or in combination therapy with radiotherapy or chemotherapy. This combination therapy is important since it exerts a dual effect: (1) the decrease of harmful side-effects mediated by chemotherapy/radiotherapy, and (2) the promotion of a synergic anticancer effect. This means that the use of aprepitant is a promising antitumor strategy, irrespectively of the tumor type to be treated, because aprepitant in many different cancer types blocks all the above-mentioned actions mediated by the undecapeptide substance P that acts as a universal mitogenic agent in cancer cells, and because tumor cells overexpress the neurokinin-1 receptor when compared with normal cells.

Antitumor strategies to treat melanoma [8] and hepatocellular carcinoma [11], as well as the clinical value of circulating microRNAs in pancreatic ductal adenocarcinoma [10] are also included in this Special Issue. Yang and co-workers from the Wenzhou-Kean University (Wenzhou, China) reviewed melanoma, which is characterized by its resistance to anticancer treatments, non-apoptotic cancer cell death mechanisms (cuproptosis, necroptosis, ferroptosis, pyroptosis), their crosstalk with apoptosis and autophagy, and the signaling pathways involved [8]. The authors discussed potential and promising therapeutic strategies favoring non-apoptotic cancer cell death for the treatment of drug-resistant melanoma. The mentioned forms of non-apoptotic cancer cell death overcome drug resistance, although the molecular processes involved must be elucidated to develop new and more specific anticancer strategies against melanoma. Li and colleagues from the Zhejiang Sci-Tch University (Hangzhou, China) have studied the antitumor action of oncolytic vaccinia viruses harboring marine lectins (e.g., *Aphrocalliste vastus* lectin) against human hepatocellular carcinoma cells [11]. The cytotoxicity of oncolytic vaccinia virus–lectins was mediated by apoptotic mechanisms in these cells and virus replication was improved when oncolytic vaccinia viruses were armed with lectins. The authors also studied the signaling pathways involved in this replication. For example, *Aphrocalliste vastus* lectin mediated androgen, lipid metabolism, phosphatidylinositol 3-kinase, Hippo, and mitogen-activated protein kinase signaling pathways via AMP-activated protein kinase crosstalk, promoting virus replication. The study showed the potential applications of oncolytic vaccinia viruses armed with lectins to fight hepatocellular carcinoma. Finally, Wnuk and colleagues from the Medical University of Silesia (Katowice, Poland) reviewed, following the PRISMA guidelines, the clinical importance of plasma/serum microRNAs for the diagnosis, prognosis, screening, and monitoring therapy of pancreatic ductal adenocarcinoma [10]. They concluded that the screening value of circulating microRNAs is uncertain and hence more research is required; that a standardization of the diagnostic methods for the expression of microRNAs is needed, since this will allow for a more precise prognosis and diagnosis; that the introduction of other biomarkers (e.g., cytokines, proteins) into microRNA diagnostic panels will increase the accuracy of the diagnostic, and that large-scale and prospective studies are needed to better understand the predictive/therapy monitoring utility of circulating microRNAs.

This Special Issue showcases several examples of promising antitumor therapeutic strategies that deserve to be developed, as well as strategies to decrease the harmful side effects promoted by chemotherapy or radiotherapy. The results reported greatly increase the knowledge about potential anticancer compounds exerting apoptotic, antiproliferative, antimetastatic, and antiangiogenic effects, and open the possibility for combination therapy utilizing some of these compounds along with chemotherapy or radiotherapy; this combination strategy would increase the antitumor efficacy and, in addition, the harmful side effects would be greatly decreased. This Special Issue also opens the door to developing promising molecular targets, to blocking tumor development, and to developing new compounds capable of specifically destroying tumor cells. The new anticancer strategies reported in this Special Issue must serve to improve the diagnosis and treatment of tumors and to increase the cure rate and quality of life of cancer patients. In sum, this Special Issue opens new promising lines of research and therapeutic possibilities to improve cancer diagnosis and treatment and shows new possibilities for translational research.
